# Supplementary material for: Promoted Mid-Infrared Photodetection of PbSe Film by Iodine Sensitization Based on Chemical Bath Deposition
Source: Nanomaterials (Basel). 2022 Apr 19;12(9):1391. doi: 10.3390/nano12091391 (PMC9105836; doi:10.3390/nano12091391)
Supplement: Supplementary file 1 [file nanomaterials-12-01391-s001.zip › nanomaterials-1673405-supplementary.pdf]

*nanomaterials*

*Supplementary Materials*

# Promoted Mid-Infrared Photodetection of PbSe Film by Iodine Sensitization Based on Chemical Bath Deposition

**Silu Peng [1], Haojie Li [1], Chaoyi Zhang [1], Jiayue Han [1], Xingchao Zhang [1], Hongxi Zhou [1,*], Xianchao Liu [1] and Jun Wang [1,2,*]**

[1] School of Optoelectronic Science and Engineering, University of Electronic Science and Technology of China, Chengdu, 610054, China; pengsilu0505@163.com (S.P.), lhj_uestc@163.com (H.L.), zhangcy1009@sina.com (C.Z.), hanjiahue_uestc@163.com (J.H.), azhangxingchao@163.com (X.Z.), liuxc@uestc.edu.cn (X.L.)
[2] State Key Laboratory of Electronic Thin Films and Integrated Devices, University of Electronic Science and Technology of China, Chengdu 610054, China.
* Correspondence: zhouhx@uestc.edu.cn (H.Z.), wjun@uestc.edu.cn (J.W.)

**Keywords:** Chemical bath deposition; PbSe; iodine sensitization; photodetector

## 1. Schematic Diagram of the Synthesis Process of $Na_2SeSO_3$.

As a Se precursor, a stock of sodium selenosulfate ($Na_2SeSO_3$), was prepared by refluxing 6g of selenium powder (Aladdin, 80 mesh, 99.999% trace metals basis) with 31.5 g of $Na_2SO_3$ (Aladdin, AR, 98%) in the 500 mL deionized water and stirred at 70°C for 6 h. Then, it was filtered to remove non-reacted selenium powder and was stored at 4°C.

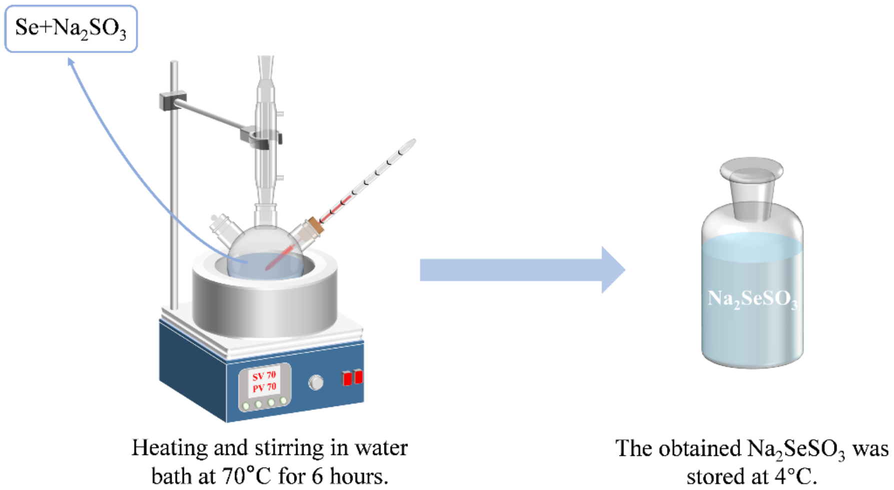


**Figure S1.** Schematic diagram of the synthesis process of $Na_2SeSO_3$.

## 2. Refined Structural Parameters for PbSe.

To further confirm the phases of the as-prepared samples, Rietveld refinements were performed on the XRD patterns of the as-prepared PbSe films using GSAS software. Detailed refinement results can be found in Table S1. The values of merit, namely, Rp, Rwp, and $\chi^2$ for PbSe-T10, PbSe-T20, and PbSe-T30, were found to be 0.0284, 0.0418, 0.3510; 0.0377, 0.0265, 0.2994, 0.0343, 0.0567, and 0.4552, respectively. This low value of the reliability factor indicates the accuracy of the structure analysis.

**Table S1.** Refined structural parameters for PbSe with the Fm-3m space groups.

| | PbSe-T10 | PbSe-T20 | PbSe-T30 |
|---|---|---|---|
| a(Å) | 6.0415(57) | 6.0773(16) | 6.1247(51) |
| b(Å) | 6.0415(57) | 6.0773(16) | 6.1247(51) |
| c(Å) | 6.0415(57) | 6.0773(16) | 6.1247(51) |
| $R_P$ | 0.0284 | 0.0377 | 0.0343 |
| $R_{WP}$ | 0.0418 | 0.0265 | 0.0567 |
| $\chi^2$ | 0.3510 | 0.2994 | 0.4552 |

## 3. The XPS Survey Spectrum of PbSe-T30 and $I_2$-PbSe-T30.

The surface elements and chemical states of PbSe-T30 and $I_2$-PbSe-T30 films were examined using XPS analysis. The survey XPS spectra were shown in Figure S2a, which showed that the main constituent elements of the samples were Pb, Se, C, and O. Figure S2b showed the survey XPS spectra of $I_2$-PbSe-T30 films, which include the elements of the Pb, Se, I, C, and O.

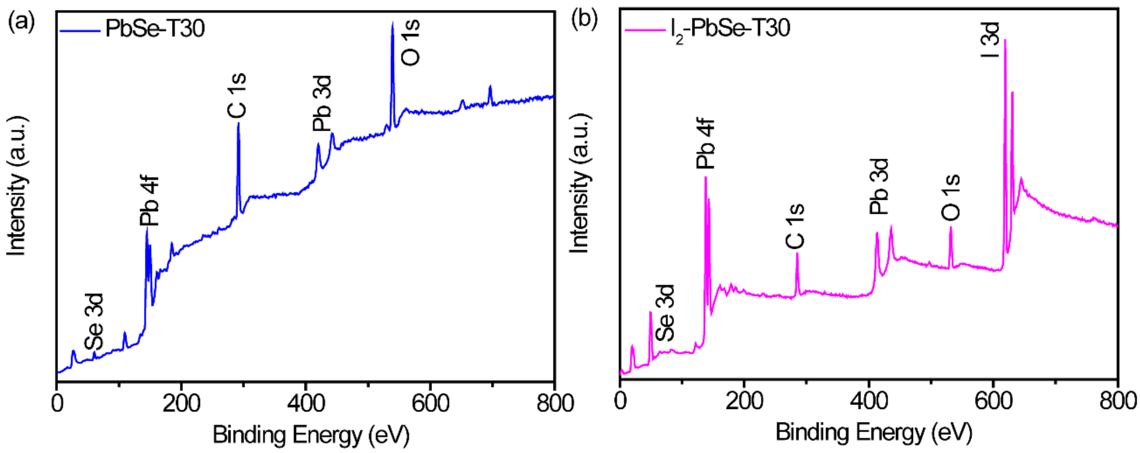


**Figure S2.** (**a**) The XPS survey spectrum of PbSe-T30; (**b**) The XPS survey spectrum of $I_2$-PbSe-T30.

## 4. The Noise Power Density as a Function of Frequency.

Figure S3 presented noise power density as a function of the frequency of PbSe-T30 and $I_2$-PbSe-T30, in which the noise has been obviously optimized for PbSe-T30 after annealing treatment. According to the obtained value ($1.91 \times 10^{-19}$ $A^2$/Hz) from the noise power spectrum at a reverse bias of 1 V and the frequency of 1Hz, the above results show that annealing sensitization treatment can not only effectively reduce the dark current and noise of the device, but can also increase its photocurrent.

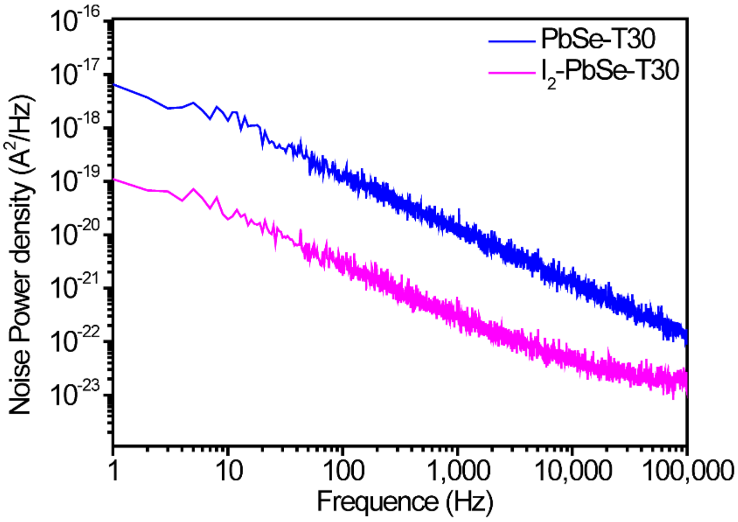


**Figure S3.** The Noise power density as a function of frequency in PbSe-T30 and $I_2$-PbSe-T30 measured at 1 V.

## 5. Photocurrent Response of $I_2$-PbSe-T30 at Different Wavelength.

Figure S4a displayed a histogram of the photocurrent value of the $I_2$-PbSe-T30 device from 808 nm to near infrared waveband (1550 nm) driven under an optical power density of 50 mW/cm$^2$ at 1 V. What's more, Figure S4b also shows the I-T curve of $I_2$-PbSe-T30 from 2250 to 5000 nm, indicating its detection capability in the mid-infrared band. It can be seen that $I_2$-PbSe-T30 has a good responsiveness at near 808 nm, and its response band width can be continued to the mid infrared region.

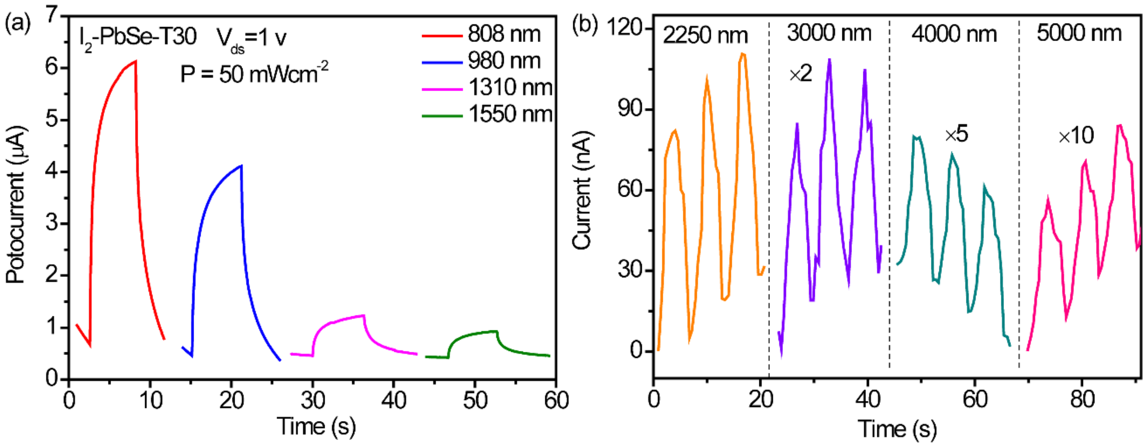


**Figure S4.** Photocurrent response of $I_2$-PbSe-T30 at different wavelength: (**a**) 808 to 1550 nm; (**b**) 2250 to 5000 nm.

## 6. Current-Voltage (I-V) Curves of $I_2$-PbSe-T30.

Figure S5 displayed the corresponding I–V curves under dark conditions and irradiation from a 808–1550 nm light source. We can see that the $I_2$-PbSe-T30 device presents the prominent photocurrent response in comparison with the dark current.

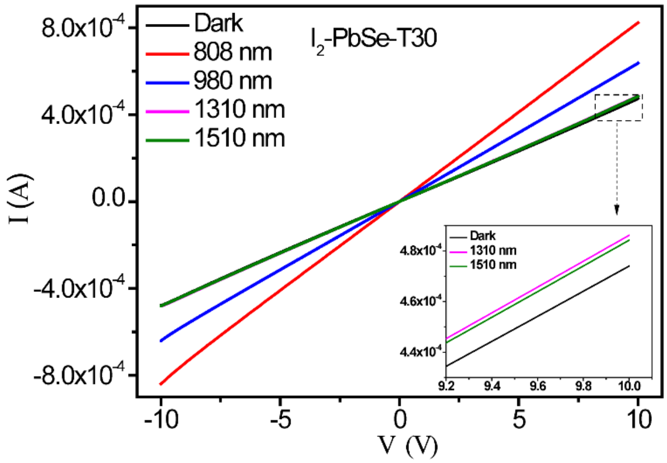


**Figure S5.** Current-voltage (I–V) curves of $I_2$-PbSe-T30 under dark and different wavelength from 808 to 1550nm.

## 7. The Plots of Photocurrent Versus Time of $I_2$-PbSe-T30.

Plots of photocurrent versus time of $I_2$-PbSe-T30 with the laser turned on and off at an applied bias of 1V from 808 to 1550 nm with different incident optical power are shown in Figure S5.

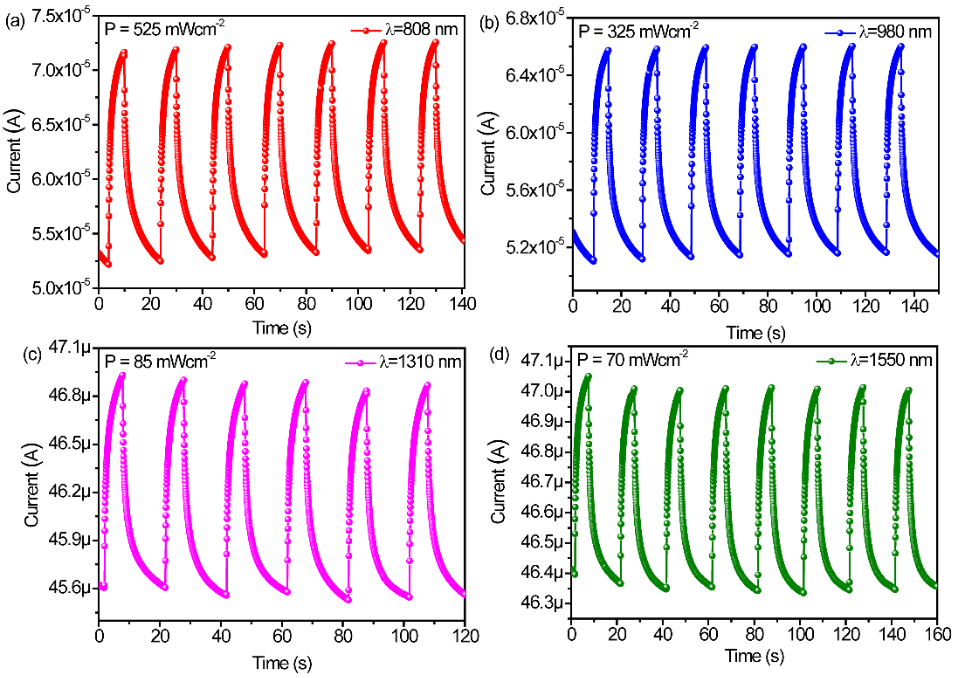


**Figure S6.** (**a**) Photocurrent as a function of time with the laser turned on and off of $I_2$-PbSe-T30 at an applied bias of 1 V from 808 to 1550 nm: (**a**) 808 nm with an incident optical power of 525 mwcm$^{-2}$; (**b**) 980 nm with an incident optical power of 325mwcm$^{-2}$; (**c**) 1310 nm with an incident optical power of 85 mwcm$^{-2}$; (**d**) 1550 nm with an incident optical power of 70 mwcm$^{-2}$.
